# The epigenetic clock and objectively measured sedentary and walking behavior in older adults: the Lothian Birth Cohort 1936

**DOI:** 10.1186/s13148-017-0438-z

**Published:** 2018-01-08

**Authors:** Catharine R. Gale, Riccardo E. Marioni, Iva Čukić, Sebastien F. Chastin, Philippa M. Dall, Manon L. Dontje, Dawn A. Skelton, Ian J. Deary, on behalf of the Seniors USP Team

**Affiliations:** 1 Centre for Cognitive Ageing & Cognitive Epidemiology, Department of Psychology, University of Edinburgh, Edinburgh, UK; 2 MRC Lifecourse Epidemiology Unit, University of Southampton, Southampton, UK; 3 Centre for Genomic and Experimental Medicine, University of Edinburgh, Edinburgh, UK; 4 Institute for Applied Health Research, School of Health and Life Sciences, Glasgow Caledonian University, Glasgow, UK; 5 Department of Movement and Sports Sciences, Faculty of Medicine and Health Sciences, Ghent University, Ghent, Belgium; 6 School of Population and Global Health, University of Western Australia, Perth, Australia

**Keywords:** Sedentary behavior, Physical activity, Aging, Epigenetic age

## Abstract

**Background:**

Estimates of biological age derived from DNA-methylation patterns—known as the epigenetic clock—are associated with mortality, physical and cognitive function, and frailty, but little is known about their relationship with sedentary behavior or physical activity. We investigated the cross-sectional relationship between two such estimates of biological age and objectively measured sedentary and walking behavior in older people.

**Methods:**

Participants were 248 members of the Lothian Birth Cohort 1936. At age 79 years, sedentary behavior and physical activity were measured over 7 days using an activPAL activity monitor. Biological age was estimated using two measures of DNA methylation-based age acceleration—i.e., extrinsic and intrinsic epigenetic age acceleration. We used linear regression to assess the relationship between these two estimates of biological age and average daily time spent sedentary, number of sit-to-stand transitions, and step count.

**Results:**

Of the six associations examined, only two were statistically significant in initial models adjusted for age and sex alone. Greater extrinsic age acceleration was associated with taking fewer steps (regression coefficient (95% CI) − 0.100 (− 0.008, − 0.001), and greater intrinsic age acceleration was associated with making more sit-to-stand transitions (regression coefficient (95% CI) 0.006 (0.0001, 0.012). When we controlled for multiple statistical testing, neither of these associations survived correction (both *P* ≥ 0.17).

**Conclusion:**

In this cross-sectional study of 79-year-olds, we found no convincing evidence that biological age, as indexed by extrinsic or intrinsic epigenetic age acceleration, was associated with objectively measured sedentary or walking behavior.

## Introduction

Prolonged sitting can increase risk of mortality and morbidity [1, 2]. Being physically active lowers the risk of dying prematurely, but the effects of sedentary behavior seem to be independent of physical activity [1]. In a systematic review, nearly 60% of those aged ≥ 60 reported sitting for ≥ 4 h a day, while in a survey where sedentary behavior was measured objectively, 67% were sedentary for > 8.5 h per day [3, 4].

Whether the amount of time spent sedentary or physically active is related to aging at a molecular level is unclear. There is consistent evidence that chronological age is strongly associated with deoxyribonucleic acid (DNA) methylation patterns [5, 6], and several DNA methylation-based biomarkers are now used to estimate biological or “epigenetic age” [7, 8]. Such measures—often referred to as the “epigenetic clock”—are predictive of mortality independent of chronological age and other risk factors, supporting the notion that they capture some aspect of biological aging [9, 10]. A study by Quach et al. investigated associations between self-reported physical activity and two of these blood-based measures of epigenetic age [11]. The proportion of immune blood cell types changes with age [12, 13]. These two epigenetic age measures were originally designed to assess whether including information about blood cell composition improved their ability to predict mortality [9]. One of these measures, known as intrinsic epigenetic age acceleration (IEAA), is independent of age-related changes in blood cell composition, and the other, known as extrinsic epigenetic age acceleration (EEAA), incorporates age-related changes in blood cell composition [11]. Quach et al. found a small correlation between being biologically older as measured by EEAA and being physically inactive, but in a meta-analysis of estimates from different ethnic groups, adjusted for other lifestyle and sociodemographic factors, the *P* value for this association was 0.09. No significant association was found between IEAA and physical activity [11]. Limitations of this study were the lack of information on sedentary behavior and the use of self-reported physical activity data which could be subject to recall error and social desirability bias.

We used data from the Lothian Birth Cohort 1936 to investigate the cross-sectional relationship between blood-based measures of epigenetic age—the epigenetic clock—and objectively measured sedentary behavior and physical activity at age 79. Our aim was to examine whether being biologically older, as estimated by epigenetic age acceleration measures, is associated with being more sedentary and taking fewer steps.

## Methods

### Participants

The Lothian Birth Cohort 1936 (LBC1936) was set up principally to study cognitive aging [14, 15]. In total, 1091 community-dwelling people were recruited at a mean age of 70 years (standard deviation (SD) 0.83). This study uses data from Wave 4. The mean age of the participants was 79 years (SD 0.45). Descriptive data on the characteristics of the sample are given in Table 1.

**Table 1:** Characteristics^1^ of the participants (*n* = 248)

Characteristics	Mean (SD), median (IQR), or number (%)
Age (years), mean (SD)	79.0 (0.45)
Female, number (%)	122 (47.1)
Percent of waking time spent sedentary, mean (SD)	62.8 (10.4)
Average no. of steps per day, median (IQR)	6509 (4945–8662)
Average no. of sit-to-stand transitions per day, median (IQR)	43.2 (35.3–51.2)
Number of chronic physical illnesses, median (IQR)	2 (1–3)
Body mass index (kg/m^2^), mean (SD)	27.3 (4.31)
Depression score, median (IQR)	1 (0–2)
Epigenetic clock measures (years), mean (SD)	
Extrinsic epigenetic age acceleration	3.16 (6.54)
Intrinsic epigenetic age acceleration	1.12 (5.35)

Depression score is based on the depression subscale of the Hospital Anxiety and Depression Scale; one item, “I feel as if I’m slowed up,” was excluded to avoid potential overlap with accelerometry measures

^1^*SD* standard deviation, *IQR* interquartile range

### Measures

#### Objective measures of sedentary and walking behavior

Consecutive participants in the Wave 4 survey of LBC1936 were invited to take part in a sub-study on sedentary behavior and physical activity until the target sample size of 300 was achieved. Using data on effect sizes found in previous studies of potential determinants of sedentary behavior [16], we carried out a series of power calculations based on 5% significance levels and 80% power and estimated that a sample size of 300 would be sufficient to detect weak to moderate associations with the relatively common risk factors that we planned to investigate. Sedentary behavior and physical activity were measured using the activPAL monitor (activPAL3c, PAL Technologies Ltd., Glasgow, UK) [17, 18]. The device is small and light (53 × 35 × 7 mm; 15 g) and was worn attached to the anterior thigh of the dominant leg with a waterproof dressing. Participants were asked to wear the activPAL continuously for 7 days, including overnight and during bathing/swimming. Participants kept a diary reporting the time they fell asleep the previous night and the time they woke up for each day of monitoring. Downloaded data were categorized into sitting, standing, and walking using proprietary activPAL software (v7.2.32) with default settings. Specifically, the minimum duration of an upright (standing or walking) event to break sitting is 10 s. This default setting is the setting tested in validation studies on detection of breaks from sedentary time [19].

The outcome measures derived from the activPAL were the percentage of time spent sedentary, number of sit-to-stand transitions, and number of steps, all averaged over the 7 days.

#### Measures of epigenetic age acceleration

Details of DNA methylation measurement have been reported [20]. In brief, blood samples for methylation were taken from participants in the Wave 4 survey. DNA methylation was measured at 485,512 sites using the Illumina Methylation BeadChip array. Bisulphite-converted DNA samples were hybridized to the Infinium HumanMethylation450 array (Infinium HD Methylation protocol and Tecan robotics) and raw intensity data were background-corrected and normalized using internal controls. Beta values were generated in minfi [21]. Quality control analysis was carried out to remove probes with a low call rate (samples with < 450,000 probes detected at *P* < 0.01), low detection rate (< 95% of probes at *P* < 0.01), and low-quality samples (manual inspection of the array control probe signals). Samples with a sex mismatch based on XY probes were removed. Probes on the X and Y chromosomes were removed, leaving 450,726 autosomal probes. Full details are reported in Shah et al. [20]. The background-corrected probes were used to calculate two measures of epigenetic age. Calculation of these measures was done online at https://labs.genetics.ucla.edu/horvath/dnamage/. Full details of the methodology are provided in Chen et al. [9]. First, the epigenetic age of each participant was estimated from their blood sample in two ways, using the approaches of Horvath, based on 353 CpG sites [7], and Hannum, based on 71 CpG sites [8]. IEAA was then defined as the residuals from a linear regression analysis of Horvath’s estimate of epigenetic age on chronological age and blood immune cell counts (plasmablasts, naive and exhausted CD8+ T cells, CD4+ T cells, natural killer cells, monocytes, and granulocytes) imputed from methylation data. Full details of the two types of software tool used to estimate these blood cell counts from methylation levels are provided in Chen et al. [9]. IEAA is therefore independent of chronological age and much of the variation in blood cell composition. IEAA is intended to capture cell-intrinsic properties of the aging process. EEAA was derived by first taking Hannum’s estimate of epigenetic age and then increasing the contribution of immune cell types to this age estimate by calculating a weighted average of Hannum’s estimate of epigenetic age and three immune blood cell types known to change with age, naïve (CD45RA+CCR7+) cytotoxic T cells, exhausted (CD28-CD45RA-) cytotoxic T cells, and plasmablasts, using an approach used by Klemera and Doubal [22]. EEAA was then defined as the residuals from a linear regression analysis of the resulting epigenetic age estimate on chronological age. EEAA tracks both age-related changes in blood cell composition and intrinsic epigenetic changes [9]. Like IEAA, EEAA is independent of chronological age.

### Covariates

Based on prior literature [2, 10, 23, 24], we chose age, sex, depressive symptoms, chronic physical disease, and body mass index as potential confounding factors. Symptoms of depression were assessed using the depression subscale of the Hospital Anxiety and Scale (HADS-D) [25]. For the purposes of the current study, we calculated this subscale score based on six items only after omitting the item “I feel as if I’m slowed up” to avoid potential construct overlap with the sedentary behavior measures. Participants provided information during interview on whether they had been diagnosed with diabetes, stroke, cardiovascular disease, high blood pressure, arthritis, or cancer; we derived a variable for number of chronic physical diseases present. Height and weight were measured with a portable stadiometer and electronic scales, respectively. BMI was calculated as weight (in kilograms)/height (in meters)^2^.

### Statistical analysis

Descriptive statistics were used to examine the characteristics of the study participants. Linear regression models were used to examine relationships between percentage of time spent sedentary, number of sit-to-stand transitions and number of steps, all averaged over seven days (dependent variables), and the two epigenetic age measures. Average daily step count and average daily number of sit-to-stand transitions were log transformed to give them a normal distribution. We adjusted first for age and sex and then in addition for the other covariates. To check whether the relationships between the two epigenetic age measures and the three outcome variables might be non-linear, we repeated the linear regression analyses with the inclusion of a quadratic term (squared EEAA or squared IEAA as appropriate). Sensitivity analyses were carried out with adjustments for technical variables related to DNA methylation measurement, namely, sample plate, BeadChip, position on BeadChip array, and hybridization date. We controlled for multiple statistical testing using the false discovery rate [26]. Analyses were carried out using STATA statistical software version 14 [27].

## Results

Of 374 people invited to participate, 304 were given an activPAL and 302 returned them. We excluded 31 participants due to incomplete diary information (*n* = 7), activPAL data quality (*n* = 5), and < 7 days of activPAL data (*n* = 19). We analyzed only those who had 7 days of activPAL data, so no assumptions about wear time would have to be made. Analyses are based on 248 of these participants who had data on epigenetic age acceleration and the covariates. This sample size resulted in 80% power (alpha = 0.5) to detect a correlation of 0.176.

Table 1 describes participants’ characteristics. Participants who spent a greater percentage of time sedentary tended to have a lower average daily step count (rho = − 0.461, *P* < 0.001). Sedentary time was not associated with number of sit-to-stand transitions. Participants having a higher average daily step count tended to make more sit-to-stand transitions (rho = 0.311, *P* < 0.001). As expected, the two measures of epigenetic age acceleration were moderately correlated (*r* = 0.424, *P* < 0.001).

Table 2 shows results of the linear regression analyses. Greater EEAA was weakly associated with taking fewer steps (regression coefficient (95% CI) − 0.100 (− 0.008, − 0.001)) in age- and sex-adjusted models. This association was attenuated after further adjustment and was not significant after correction for multiple testing. EEAA was not associated with spending more time sedentary or number of sit-to-stand transitions.

**Table 2:** Results of linear regression analyses of measures of walking and sedentary behavior according to epigenetic age acceleration estimates at age 79

	Regression coefficient (95% CI) and *P* values^2^		
Epigenetic age acceleration measures, per year increase	Percent of time spent sedentary	Logged average number of steps per day	Logged average number of sit-to-stand transitions per day
Extrinsic epigenetic age acceleration			
Adjusted for sex and age	0.181 (− 0.025, 0.387) *P* = 0.085/*P* corrected = 0.187	− 0.100 (− 0.008, − 0.001) *P* = 0.027/*P* corrected = 0.149	− 0.001 (− 0.006, 0.004) *P* = 0.671/*P* corrected = 0.671
Multivariable-adjusted^1^	0.109 (− 0.093, 0.310) *P* = 0.289/*P* corrected = 0.397	− 0.007 (− 0.015, 0.001) *P* = 0.083/*P* corrected = 0.187	− 0.003 (− 0.006, 0.005) *P* = 0.922/*P* corrected = 0.920
Intrinsic epigenetic age acceleration			
Adjusted for sex and age	− 0.105 (− 0.340, 0.129) *P* = 0.379/*P* corrected = 0.429	− 0.005 (− 0.015, 0.004) *P* = 0.273/*P* corrected = 0.397	0.006 (0.0001, 0.012) *P* = 0.049/*P* corrected = 0.181
Multivariable-adjusted^1^	− 0.146 (− 0.371, 0.079) *P* = 0.203/*P* corrected = 0.372	− 0.004 (− 0.013, 0.005) *P* = 0.390/*P* corrected = 0.429	0.007 (0.001, 0.013) *P* = 0.027/*P* corrected = 0.149

^1^Adjusted for age, sex, body mass index, depression score, and number of chronic physical illnesses.

^2^*P* values are shown uncorrected and with correction for the false discovery rate

Greater IEAA was weakly associated with making more sit-to-stand transitions in both age- and sex-adjusted and multivariable-adjusted models. The multivariable-adjusted regression coefficient (95% CI) was 0.007 (0.001, 0.013). Again, these associations were non-significant after correction for multiple testing. There were no significant associations between IEAA and sedentary time or number of steps. Further adjustment for technical variables related to DNA methylation measurement (sample plate, BeadChip array, position on BeadChip array and hybridization date) did not change these estimates.

We re-ran these models with the addition of a quadratic term (squared EEAA or squared IEAA as appropriate) to check whether the relationships between EEAA or IEAA and the three outcomes might be non-linear. The quadratic term was not statistically significant in any of the models.

## Discussion

These findings suggest that the amount of time older people spend being sedentary or physically active is not related to their biological age as estimated by the epigenetic clock. A previous study of over 4000 people found a weak significant correlation (*r* = − 0.07, adjusted for ethnicity and dataset) between greater EEAA and being physically inactive in pooled samples from the Women’s Health Initiative [11]. In a meta-analysis of estimates from different ethnic groups that included additional data from the InChianti cohort and adjustment for other lifestyle and sociodemographic factors, the association between EEAA and physical inactivity was attenuated (*P* = 0.09) [11]. No association was found with IEAA [11]. Our study was underpowered to detect a correlation as small as that observed in this latter study, but it is worth noting that the estimate of the size of the effect between EEAA and physical inactivity in that study may be conservative due to inaccuracies in the self-reported activity data.

Here, we measured sedentary and walking behavior objectively using an activPAL and based our outcomes on 7 days of data. It is of course possible that wearing the activPAL device led some participants to change their behavior, but recent evidence suggests that such change tends to be limited to the first day of wearing the device [28]. Under our study protocol, the first day of wearing the device is a half day, and data from half days were not used for analysis. This makes it more likely that the data we obtained from the activPAL is a reflection of usual behavior. Studies have varied widely in the length of time participants have been required to wear activity monitors, but according to most best practice guidelines [29], 7 days is an appropriate period to obtain a representative sample of typical behavior.

Blood-based measures of epigenetic age have been shown to be associated with mortality [9, 10, 30], physical and cognitive fitness [31], and frailty [32]. Little is known about the relationship between these biomarkers and physical activity or sedentary behavior. Our findings suggest that EEAA or IEAA is not associated with objectively measured sedentary or walking behavior in older people.

## Abbreviations

BMI: Body mass index
DNA: Deoxyribonucleic acid
EEAA: Extrinsic epigenetic age acceleration
IEAA: Intrinsic epigenetic age acceleration
IQR: Interquartile range
SD: Standard deviation
